# Supplementary material for: Convergent evolution of viral-like Borg archaeal extrachromosomal elements and giant eukaryotic viruses
Source: Nat Commun. 2025 Nov 27;16:10641. doi: 10.1038/s41467-025-65646-7 (PMC12660748; doi:10.1038/s41467-025-65646-7)
Supplement: Supplementary file 1 — Supplemtary Information [file 41467_2025_65646_MOESM1_ESM.pdf]

SUPPLEMENTARY INFORMATION

Convergent evolution of viral-like Borg archaeal extrachromosomal elements and giant eukaryotic viruses

Banfield et al.

The Supplementary Information relates to the Results section

Table of contents **Page number**

Supplementary Text	2-8
Supplementary Figures	9-22
Supplementary References	23

Supplementary Text

DeSAMPylases

The presence of the nucleophile serine in the active site region distinguishes metalloproteases from cysteine proteases that have the catalytic triad of cysteine and nearby histidine and aspartate residues. Interestingly, a *Mycobacterium ulcerans* protein annotated as a CysO-cysteine peptidase (AF-X8F1Y7) shares the active site residues of the putative de-SAMP metalloproteases and aligns, for example, with Orange 478 and cMp 650 putative deSAMPylases. The *M. ulcerans* protein is likely not a cysteine peptidase. We conclude that deSAMP-like metalloproteases analogous to those of Borgs and *Methanoperedens* also exist in these bacteria. All putative deSAMPylases tested bind Zn into the active site with high confidence.

Structural and potential functional diversity across DMPS clades

Borg and related protein DPMS clades

The putative protein sequences from Borgs (subfam0199), the complete *Methanoperedens* genome (cMp) and the most closely related sequences from NCBI were analyzed phylogenetically. NCBI sequences are from other *Methanoperedens*, some methanogens, Thermococcales, Bathyarchaeia, Thermoplasmatales, some DPANN and unclassified archaea, and many sequences are from Chloroflexi. Chloroflexi proteins have been repeatedly observed to place phylogenetically with those of archaea (e.g., (Hug et al. 2013), and vice versa. The pattern supports extensive gene transfer between Chloroflexi and *Methanoperedens*.

Structural variation

Representative sequences from Borg and *Methanoperedens* clades were aligned to each other and 5mm0, as well as to other PDB models for proteins with the same DPMS function. All align closely in the cytoplasmic component of reference PDB structures and some align very closely over the entire protein (**Supplementary Figure 3B)**. Some sequences completely lack the membrane anchor region (yet are still profiled as DPMS; **Figure 3C**).Flanking genes are generally not good candidates for a separate membrane subunit that could form a complex representing the complete protein. Many AlfaFold DB sequences (e.g., *Methanocaldococcus jannaschii* MJ1222, many bacteria and a sequence from a viral metagenome) also lack the membrane anchor. Although some Borg proteins and a few other proteins have an extra cytoplasmic domain (**Supplementary Figure 3D**), no analogous sequences were found in public data via Foldseek or searches.

Key residues

To predict functional relatedness to the biochemically characterized type-I, -II and -III catalytic DPMS family representatives we searched for 40 expected, conserved residues reported in **Supplementary Figure 3** of (Gandini et al. 2017). These are involved in metal

binding, catalysis and substrate interactions. Residues were considered comparable if they have biochemical characteristics similar to those identified in the experimental studies. Some proteins, such as Cobalt Borg 211 have all residues as expected based on 5mm0 and the acceptor loops of the Borg protein and 5mm0 are clearly aligned (**Figure 3A**), but most differed in some way from the references. Given the huge phylogenetic spread of proteins under consideration, for the final analysis, we prioritized the DxDxQ of the DADLQ region of 5mm0 that is required for metal binding (particularly, the 2nd D and Q (or similar).

Potential functional diversity and evolution

Presence/absence of the membrane anchor and the presence of residues of the expected metal binding motif were mapped onto the phylogenetic tree (**Figure 3**, **Supplementary Data 5**). Clade 1 is comprised of 9 Borg proteins that include the anchor region and all have the DxDxQ residues. Clade 2, sibling to Clade 1, is comprised of sequences from diverse archaea, two sequences from *Methanoperedens*, as well as sequences from Thermoplasmatales, Bathyarchaeia, Chloroflexi, and a single Purple Borg sequence. The proteins possess the cytoplasmic domain and the membrane anchor, with the exception of a sub-clade that includes one Chloroflexi, one *Methanoperedens* and two Borg sequences that lack the membrane anchor. The placement within a much larger clade of proteins with the membrane anchor suggests these anchor-free variants derived from an ancestral protein with an anchor. All Clade 2 sequences have the DxDxQ residues, except one sequence from Purple Borg, which has NxDxQ, and is likely able to bind the metal.

Clades 1 and 2, along with one sequence from Iris Borg and seven sequences from various archaea all have the capacity to bind the metal, and represent a major subdivision in the tree.

Clade 3 is comprised of 6 Borg sequences and a single sequence from the cMp genome. All proteins lack the membrane region and lack the expected glutamine residue. Instead, these proteins have S, N or A. Of these, only those with N instead of Q may bind the metal.

Clade 4 is comprised of 10 Borg sequences, all of which lack the membrane region and all lack the DxDxQ residues, with S in place of Q, as for a subset of sequences in Clade 3.

Clade 5 is comprised of 5 Borg proteins that lack the membrane region and with DxDxT instead of DxDxQ. Closely related is Viridian 228, which features a membrane anchor, has T in place of Q.

Clade 6 is comprised of 8 Borg proteins and one protein from the cMp genome. All possess the membrane region and, like Clade 5, have T in place of Q.

Clade 7 is comprised of numerous database sequences from *Methanoperedens* and other ANME archaea (collapsed), 19 Borg sequences that form a sibling sub-clade and two more distantly related cMp sequences.

Clade 7 also contains two partial sequences from Brown Borg (377 - 378) that represent a split protein. The genome is fully supported by reads, thus this is not an assembly error. The gene switches from +2 to +3 frame and 377 has a short C-terminal extension (**Supplementary Figure 7**). A +1 frame shift follows a "slippery" polyT (and generally AT-rich) region, as is often the case for frameshifted genes. Assuming frameshifting leads to a complete protein, all Clade 7 representatives have the membrane anchor and the expected DxDxQ motif. Basal to Clade 7 Borg are two Methanoperedens cMp proteins with the membrane anchor and the expected DxDxQ residues.

Clades 3-7 plus a few intervening sequences, represent the second major subdivision in the tree. Sub-clades of proteins without the membrane anchor phylogenetically intersperse with those possessing the membrane anchor. The tree topology is suggestive of evolution from a membrane anchored ancestor (as for the first major subdivision), potentially with three separate membrane anchor loss events with a single origination of the DxDxT substitution.The placement of single *Methanoperedens* proteins in Borg-dominated Clades 2 and 3 suggest that the host acquired these variants via lateral gene transfer from Borgs whereas the reverse pattern in Clade 7 is best explained by gain of Borg proteins from *Methanoperedens* or related archaea.

<u>Extra cytoplasmic domain proteins and possible multimerization</u>

The Borg, *Methanoregula* and *Methanoperedens* genomes with extra cytoplasmic domain (**Supplementary Figure 6**) are consistently profiled as DPMS. The next best annotation (Dali) is glucosyl-3-phosphoglycerate synthase (also has only one cytoplasmic domain) in which the metal binding site is formed by widely separated Asp and His (thus not resembling the DxDxQ metal binding site of DPMS).

Some Borg proteins (e.g., Cobalt and Olive), and proteins identified in *Methanoregula* and a *Methanoperedens* genomes with an extra cytoplasmic domain form homotetramers based on alignment of the hydrophobic regions (**Supplementary Figure 6C**) with modest confidence. Tetramerization is not unexpected for DPMS itself, as PDB 5EKE is known to tetramerize (and experimental methods would have inhibited detection of multimers in several other structure characterization studies, e.g., 5mm0).

All seven proteins with an extra cytoplasmic domain contain metal binding sites in the main cytoplasmic subunit and in two cases, the DxDxQ motif also occurs in the additional cytoplasmic subunit. In the Cobalt Borg protein, the N in the Q site could enable metal binding. In four cases, the first cytoplasmic domain has D/ExDxR/K, but the activation loops are either not present, or additional loops contribute bulky aromatics to the active site that preclude access of the GDP-Mannose donor substrate, suggesting that they may have a function other than mannose donation to a substrate.

Gene clusters largely implicated in surface modification

In addition to DPMS, Borgs encode many multicopy genes apparently involved in production of extracellular polymers and glycosylation of extracellular proteins and/or lipids and nucleotide sugar transformations. Prominent are, for example, UDP-sugar-epimerases (subfam1264), including UDP-N-acetylglucosamine 4-epimerase, UDP-Glucuronic acid 4-epimerase, GDP-mannose-3', 5' -epimerase. These are structurally similar. For example, the six Cobalt Borg subfam1264 proteins all align to each other (despite slightly different PDB-based annotations) and likely play a role in production of glycoproteins, glycolipids, or proteoglycans. Also prevalent are proteins for dTDP-L-rhamnose biosynthesis (e.g., glucose-1-phosphate thymidyltransferase, subfam2340) and glycosyl/mannosyl transferases (subfam515, subfam1659, subfam1055).

Within the region depicted in **Figure 4**, Cobalt 305 appears to have genes for synthesis of glucosamine-6P, a precursor of N-acetylglucosamine. Nine genes encode proteins that likely perform reactions involving N-acetylglucosamine (GlcNAc), N-acetylmannosamine (ManNAc), uridine diphosphate N-acetylglucosamine (UDP-GlcNAc), UDP-3-oxo-GlcNAc and UDP-N-acetylmannosamine (UDP-ManNAc). For example, Cobalt 286 is predicted to be (UDP-GlcNAc) 2-epimerase, which interconverts UDP-GlcNAc to UDP-ManNAc, and 298 UDP-N-acetylglucosamine 4-epimerase converts UDP-GlcNAc to UDP-GalNAc. 287 is likely chitin deacetylase (chitin is a polymer of GlcNAc). A putative UDP-ManNAc dehydrogenase may provide UDP-N-acetyl-alpha-D-mannosaminouronate for biosynthesis of sialic acid-like polymers. Intriguingly, many genes in this region of the Cobalt Borg genome (some sequentially encoded) appear to encode the glycosyltransferase MshA), one of the most prevalent multicopy proteins (subfam1055, **Figure 1A**) and the first step in mycothiol biosynthesis. Encoded in close proximity to MshA and DPMS are deacetylases that may function as the deacetylase MshB (some have what appear to be accessory carbohydrate-binding domains). The ligase (MshC) and acetylation (MshD) genes were not identified.

Within the Cobalt Borg genome region shown in **Figure 4** is a cluster of four genes (308-311) with structures indicative of biosynthesis of O-methyl phosphoramidate (MeOPN), an unusual bacterial capsular polysaccharide (*78*). Cobalt 309, likely L-glutamine kinase, catalyzes the ATP-dependent phosphorylation of the amide nitrogen of L-glutamine to form L-glutamine phosphate, the first committed step in O-methyl phosphoramidate (MeOPN) biosynthesis. 308 then may convert CTP + L-glutamine phosphate to CDP-L-glutamine, 310 may convert CDP-L-glutamine to L-glutamate and cytidine diphosphoramidate. 311 has several possible annotations, but the best annotated Foldseek hit (e-27 and with good structural alignment) suggests CTP:phosphoglutamine cytidylyltransferase, which is also involved in the biosynthesis of the O-methyl phosphoramidate (MeOPN). CTP:phosphoglutamine cytidylyltransferase may displace the pyrophosphate from a nucleoside triphosphate by phosphoramidate to generate the nucleoside diphosphoramidate (these enzymes correspond to Cj1418, 1417, 1416 of (Taylor et al. 2017).

Cobalt 308 is also a CTP:phosphoglutamine cytidylyltransferase, but with an N-terminal

PEP-mutase domain, which is notable in that this enzyme can form C-P bonds that are the hallmark of phosphonates. Cobalt 319 encodes a protein structurally very similar to a fusion phosphonate-specific cytidylyltransferase and 2-aminoethylphosphonate (AEP) transaminase, thus also likely involved in phosphonate biosynthesis.

The genomes appear to encode many proteins involved in phospholipid biosynthesis. UDP-GlcNAc 3-dehydrogenase (GnnA) is involved in lipid A production (β-(1→6)-linked GlcNAc disaccharide (e.g., Cobalt 300 modeled to 7bvj). Cobalt 320 aligns in the membrane portion with a CDP-alcohol phosphotransferase that transfers a substituted phosphate group from a CDP-linked donor to an alcohol acceptor, an essential reaction for phospholipid biosynthesis. The Borg protein has the exactly conserved active site, however it lacks the N-terminal cytosolic domain. Genes in the same genomic region encode proteins with close structural similarity to Lipid II flippases, which translocate Lipid II across the bacterial cytoplasmic membrane (in bacteria, for synthesis of peptidoglycan). Predicted multicopy glycerophosphodiesterases (subfam2067, e.g., Cobalt 222-225) also may be involved in glycerophospholipid metabolism.

Histones and Vault proteins

Evidence for Borg encapsulation motivated a search for genes that may organize and package the genomic DNA. Borg genomes encode either single or multiple credible histone proteins (e.g., Green 372), histone remodeling helicases (e.g., Cobalt 739, 742), histone protein methyl/acetyl transferases, and histone protein demethyl/deacetyl transferases (e.g., Green 1225). We also identified one helicase-based protein with an ATP binding site that is known to associate with histones, but this protein has an extra nuclease NucT domain that may call into question a role for this protein in histone loading/unloading or packaging.

The Amethyst Borg genome encodes two adjacent (811, 812; **Supplementary Figure 13A**) and at least one additional histone-like protein (823), all of which have very similar structures. The adjacent encoded Amethyst histones form a dimer that aligns well with the known histone dimer configuration **(Supplementary Figure 13B)**.

Histones packaging may be facilitated by post-translational modification of the histone proteins. Acetylation factors in Borg genomes suggest that the histones can be acetylated to active transcription. Also identified was NatD (in 4u9w is bound to H4/H2A peptide and CoA). This is among the most selective N-terminal acetyltransferases (NATs); its only known substrates are histones H4 and H2A. This protein acetylates Arg3 of the eukaryotic histone. The structure is similar and it has the ASP in the expected position to bind Arg3 and a pocket that would accommodate CoA (acetylation donor) to catalyze heterochromatin formation. Amethyst Borg has a small MORC3 CW domain-like protein This zinc finger domain binds to methylated histone protein tails and recruits protein.

Borg genomes often encode what appear to be major vault proteins that were not identified in the host *Methanoperedens* genomes. Multimer predictions indicate that these assemble into portions of the expected barrel-like structures. However, computational

limitations preclude *in silico* assembly the many subunits that would be required to fully define the complex. Intriguingly, in some genomes (e.g,. Ruby) the putative vault is encoded prior to a histone and a universal single copy protein predicted to be an exonuclease (ubiquitous single copy gene of subfam0908). The subfam0908 protein is profiled as 1zbh 3'-end specific recognition of histone mRNA stem-loop by 3'-exonuclease, but just has the exonuclease RNA degrading domain. In Ochre, Orange, Green, Purple, Rose, Sky, Amber, Amethyst, Emerald, and Viridian Borg genomes, this exonuclease gene occurs in close proximity to genes encoding either the putative vault or a histone, possibly suggesting connections between their functions.

ESCRT proteins for membrane remodeling and tubulin-related proteins

It was once thought that ESCRT-III proteins, which are involved in processes such as multivesicular body formation, plasma membrane repair, nuclear envelope reformation, and viral budding (McCullough et al., 2018), were specific to Eukaryotes. However, ESCRT-III homologs occur in TACK archaea (Lindås et al. 2008; Samson et al. 2008), where they have been implicated in membrane scission during viral budding (Liu et al., 2017), extracellular vesicle formation (Ellen et al. 2009), and cell division (*82*). ESCRT-III proteins (and their regulators) are also encoded by the genomes of some Asgard archaea, supporting the proposal that the eukaryotic proteins have an archaeal origin (*83*). ESCRT III-like proteins (as well as some other ESCRT-related proteins) occur in some Borgs (**Supplementary Figure 14**). These proteins may create an invagination in the host cell lipid membrane during Borg encapsulation if they function in an analogous way to ESCRT proteins in *Nucleocytoviricota*, which have a lipid component within their capsids.

A large single copy gene (encoding a protein of 1057 ± 27 amino acids) occurs in all but one of the 17 Borgs (subfam2487) and encodes a protein profiled as similar to tubulin / FtsZ (4b45), but alignment is only partial, and involves the start and ends of the Borg proteins. Intervening between these regions of the Borg proteins is a long coiled-coil (e.g., Orange 265). This region is also present in some AFDB sequences, e.g., *Halorientalis regularis* AF-A0A1G7LXG2-F1. The *Halorientalis regularis* protein aligns reasonably well with Borg proteins (at the sequence and structure level). There are also a few examples from *Methanoperedens*.

An enigmatic, highly multicopy protein

A highly prevalent multicopy subfamily in the Borg genomes, subfam1834 with 126 members, and up to 10 copies per genome, encodes a small protein that is always found at the start and end of the genomes (with related proteins distributed throughout). It has a strongly positively charged surface and a topology that resembles that of the best (but low scoring) PDB match (4pt7), the initiator RepA, and expression of this gene was detected for up to five of the eight Black Borg genes in 50 cm, 80 cm, 90cm and 115 cm soil datasets. Some members of subfam1834 are somewhat similar to transcriptional regulators. However, co-folding with double stranded DNA using AlphaFold 3 did not predict tight association. Modeling using the alignment in place of AF2 templates

generated a similar moderate confidence structural prediction and did not uncover new functional clues.

Supplementary Figures

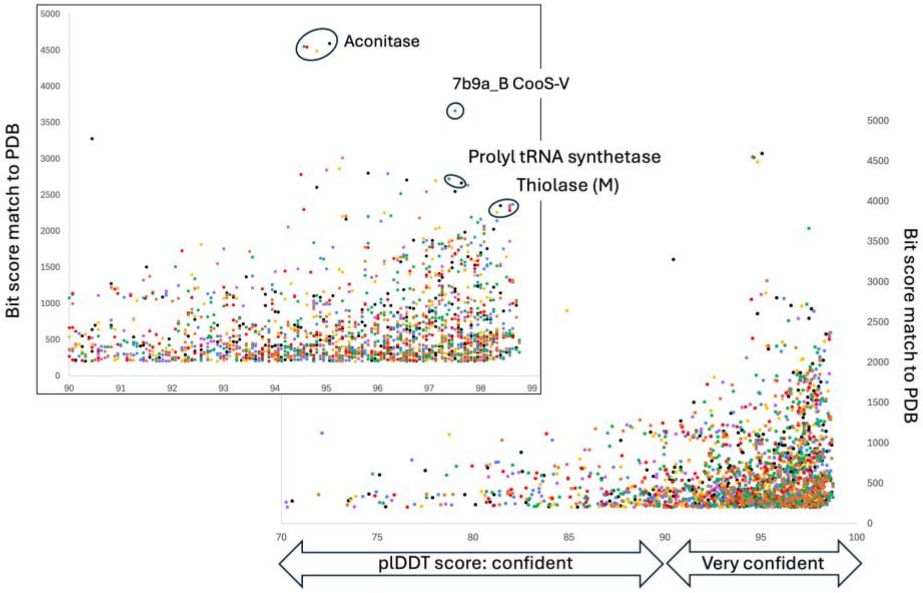

Supplementary Figure 1: The distribution of scores for proteins with confident (pLDDT > 70) or very confident (pLDDT > 90) structure predictions and bitscores to best matches in PDB of > 200, colored by Borg of origin. Insert in the upper left shows just the very confident structure predictions and the corresponding bitscore of each match in PDB, with some outliers annotated. Calculations for all proteins from 7 complete Borg genomes (9661 proteins) yielded 8847 structures (pLDDT > 27) confident or very confident structure predictions in 63% of cases. For tabulation of scores by protein and other statistics, see **Supplementary Data Tables 2 - 8**.

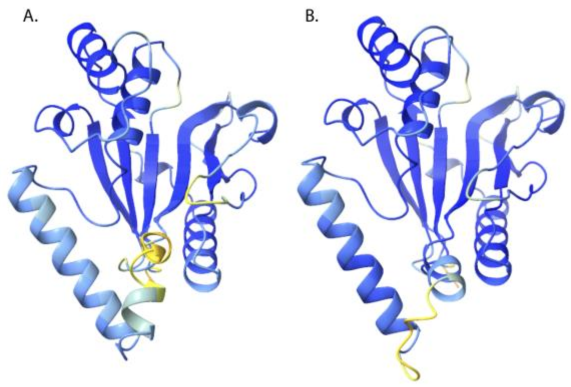

Supplementary Figure 2: Comparison of folds predicted for Orange Borg 1200, a putative deSAMPylase, A. with and B. without use of PDB templates, colored by AlfaFold pLDDT confidence (darker blue indicates higher confidence). The template-free prediction is based on an alignment of the 315 Borg protein sequences (see Figure 1B).The folds have similar fold confidence scores.

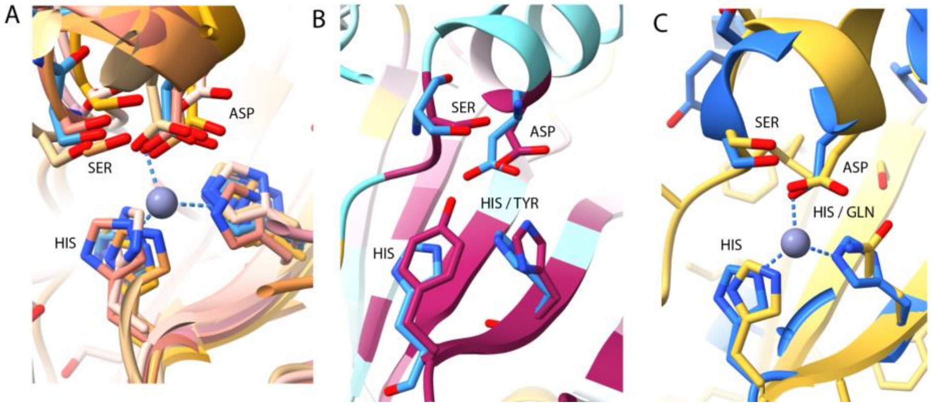

Supplementary Figure 3: A. Most of the 315 Borg proteins are likely metalloproteases with the HIS,HIS,ASP and SER active site residues, as illustrated by six examples of active site regions of six Cobalt Borg de-SAMP proteins aligned with 3rzu (Crystal Structure of the Catalytic Domain of AMSH), which is shown in blue. B. A few Borg proteins do not display these exact expected active site residues, thus may have modified functionality: Amber 1107 colored by conservation (cyan to magenta with magenta being highly conserved based upon 315 sequences), aligned to active site residues of 3rzu (blue). Note the tyrosine in place of histidine in the active site. The one Zn included in the model is predicted to localize to the active site, with global ipTM = 0.88, PTM = 0.88). **C.** Amber 1231 (gold) compared to 4msd (blue), illustrating an active site variant with glutamine in place of histidine. Zn is predicted to localize to the active site, global ipTM = 0.94, PTM = 0.81.

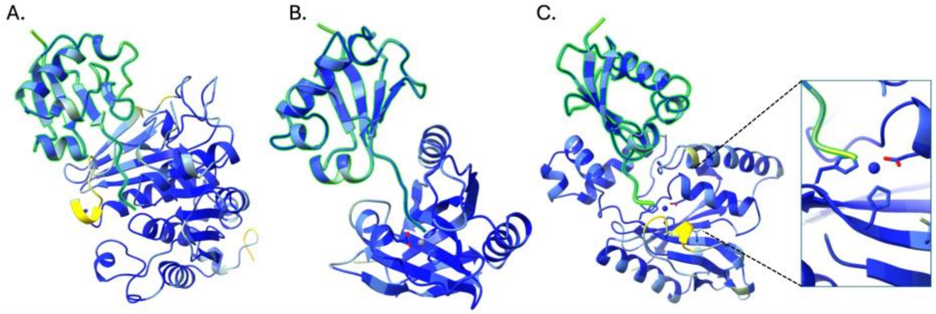

Supplementary Figure 4: AlphaFold 3 cofolding structure predictions involving SAMP (outlined); with proteins colored using AlphaFold confidence values (blue is high confidence). **A**. The *Methanoperedens* SAMP (cMp 2846) bound to a *Methanoperedens* SAMP-adding enzyme E1 (cMp 3305), ipTM = 0.86, pTM = 0.92; RMSD 0.139 - 0.191 for five model comparison). **B.** The Methanoperedens SAMP bound to a Methanoperedens deSAMPyase (650) and Zn with high confidence (ipTM = 0.85, pTM =0.86). **C.** The structure of the *Methanoperedens* SAMP bound to a putative Borg deSAMPylase (Orange 883), pTM = 0.90 pTM = 0.92). The multimers involving deSAMP display the conserved β-grasp fold. The zoomed view shows that the di-glycine tail of the SAMP engages with the His,His,Asp active site, as expected.The *Methanoperedens* proteins were identified using *Haloferax* references.

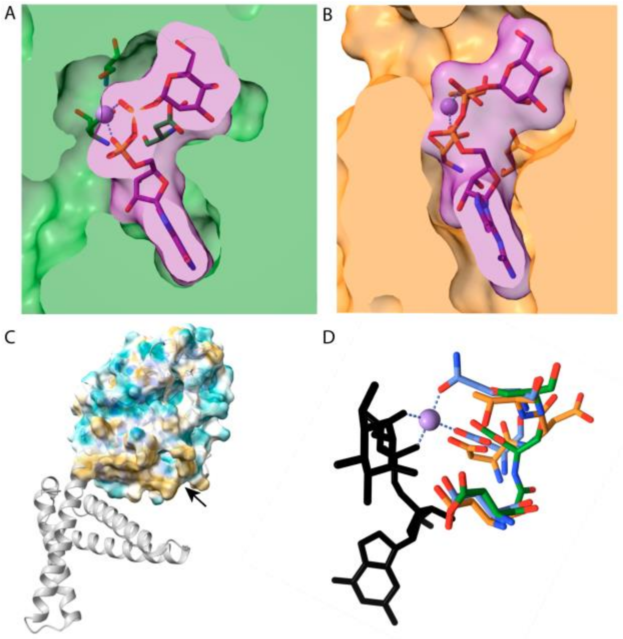

Supplementary Figure 5: Predicted features of DPMS proteins. **A.** Surface representation of the predicted active site pocket of Green 271 reveals a cavity that could accommodate the UDP-Mannose. **B.** The active site pocket of Orange 206 could accommodate the UDP-Mannose. **C.** Anchorless versions of DPMS are typically predicted to have a hydrophobic patch (gold color, see arrow) in the membrane contact region adjacent to where an anchor occurs in some variants, as shown by 5mm0 (gray). **D.** The conserved D,D and Q active site of 5mm0 (blue) with the Mn shown in purple and the ligand in black, aligned to the residues found in two Borg proteins that lack a membrane anchor and have divergent active sites (Green 271 of Clade 3 (green): S/Q, and Orange 206 of Clade 5 (orange): T/Q, see **Figure 3A**).

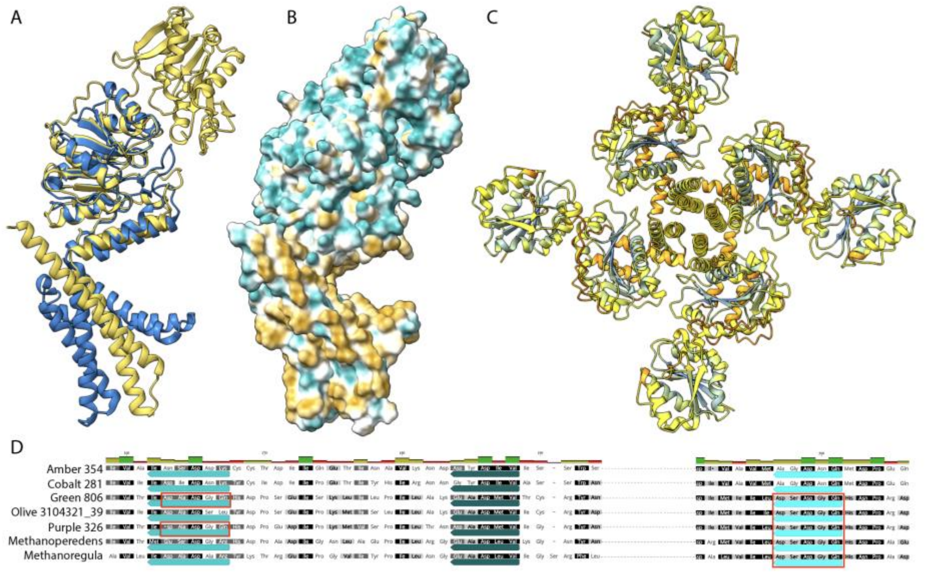

Supplementary Figure 6: Putative DPMS and variants with an extra cytoplasmic domain. **A**. Green 806 (gold) as an example of proteins with an extra cytoplasmic domain, aligned to 5mm0 (blue) **B.** Hydrophobicity of Green 806, with gold indicating hydrophobic regions that are especially prevalent in the membrane anchor region. **C**. Tetramer of Purple 326 colored by AlphaFold plDDT scores (high confidence is dark blue); buried area 1587.3 Å. Despite very modest confidence scores (ipTM 0.26 - 0.27 for all models, pTM 0.29), DPMS tetramer formation is supported by some experimental studies [21]. **D.** Regions of the multisequence alignment for examples of DPMS with extra cytoplasmic domain showing the residues of the potentially active site in the main and extra cytoplasmic domain (canonical active sites are indicated by red boxes).

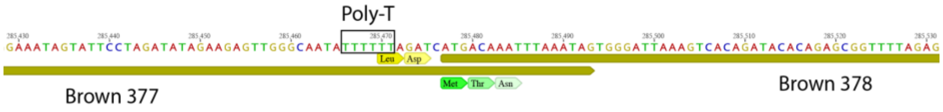

Supplementary Figure 7: One predicted apparently frameshifted DPMS gene encoding a protein with the DxDxQ motif (Brown 377,378). The genome is fully supported by reads, thus this is not an assembly error. 377 has a short C-terminal extension. The frame shift appears to occur after a "slippery" poly-T (and generally AT-rich) region, as is often the case for frame shifted genes.

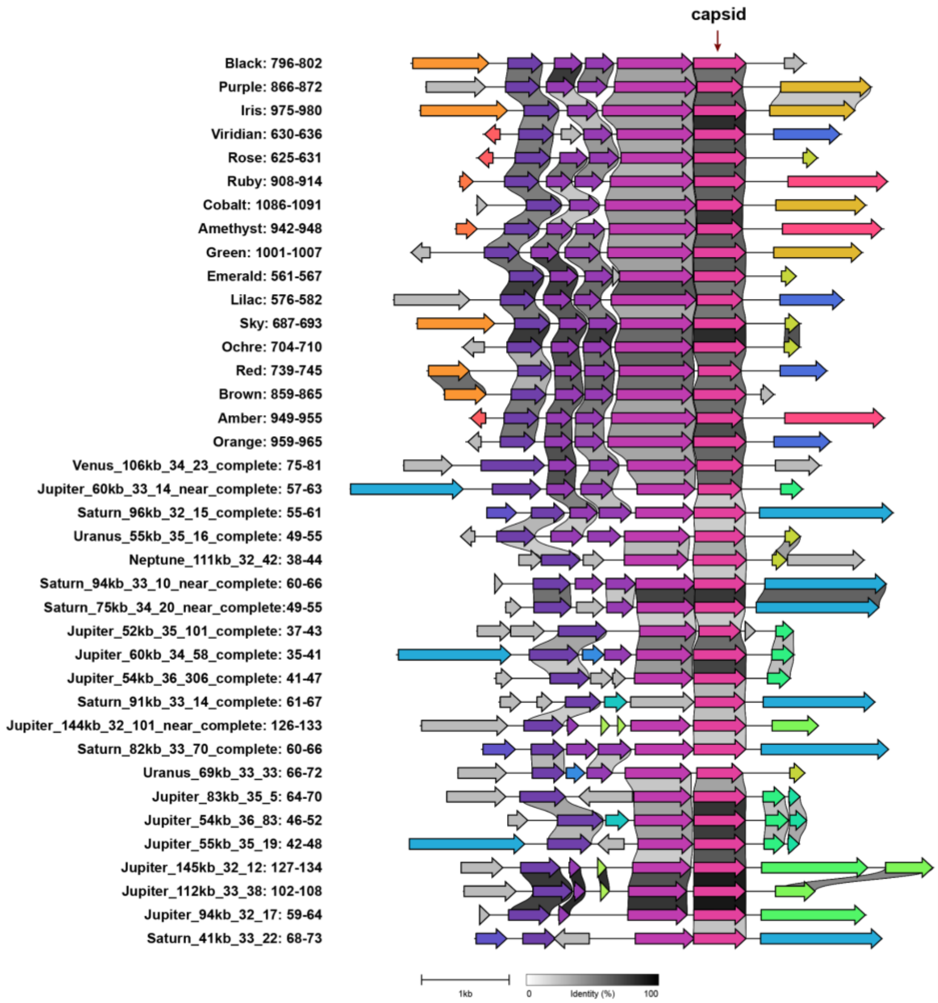

Supplementary Figure 8: Gene content and gene order conservation in Borg and mini-Borg genomes that encode a gene cluster that features a putative capsid protein (including Black 801), pink. which is highly expressed in some samples, see **Figure 5A**). The functions of the prior conserved genes could not be discerned by domain analysis, structure prediction analysis (even using the multi protein sequence alignment instead of PDB templates) or multimer calculations. When expressed by Black Borg, the genes in this region are transcribed independently yet when expressed in mini-Borgs, they form a single transcript. Genes classified as homologs share the same color.

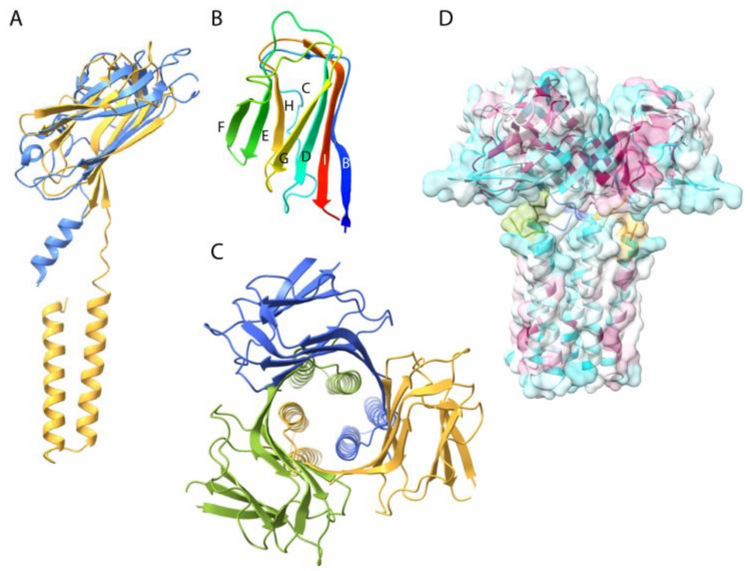

Supplementary Figure 9 The 17 Borg genomes encode multicopy (subfam1773, 43 total) capsid-like proteins, initially profiled as a bacteriophage tail knob protein (4k6b, a trimer). **A.** Sky 263 monomer (gold) aligned to 4k6b (blue). The alpha helical regions of the Borg proteins are hydrophobic, thus may insert into a membrane. The predicted structure also matches reasonably to the chain B of 6h9c (the capsid-like protein illustrated in **Figure 5** aligns with 6h9c VP7 subunit M). **B**. Detail of the beta sheet arrangements in Sky 263 indicating classification as a jelly roll fold. **C**. Sky 263 as a homo-trimer, with each subunit colored differently and viewed from above, first calculation: ipTM 0.67, pTM = 0.71, second calculation ipTM = 0.76. pTM = 0.78. Lowest confidence is localized to the alpha helical regions. Scores for the pentamer are much lower than for the trimer (ipTM = 0.25, pTM = 0.28). **D**. Side view of the Sky 263 homo-trimer colored by conservation (cyan to magenta, with magenta as high confidence) showing conserved residues at the subunit junctions in the jelly roll fold region.

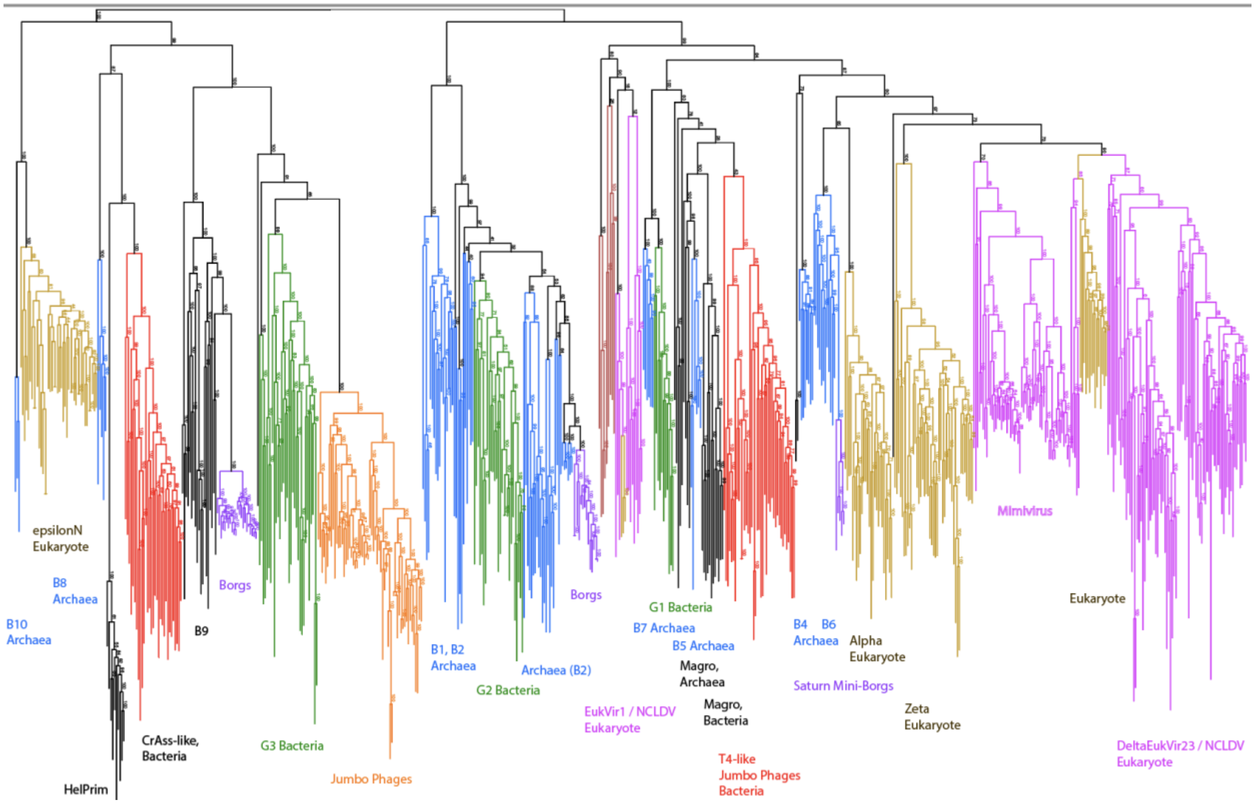

Supplementary Figure 10: DNA polymerase B tree featuring a sampling of reference sequences (with clade names) from [73] and sequences from internal databases for *Nucleocytoviricota* / mimiviruses, and Jumbo phages. Sequences that are always present in Borgs clade with B9, which Kazlauskas noted are mostly (96%) from metagenomic databases (only two sequences are annotated being from a Thermoplasmatales archaeon and a Candidatus Woesearchaeota archaeon). Other B9 sequences we identified in metagenomic data were likely Woesearchaeota or viral. Other Borg sequences fall into B2, and place with those of host *Methanoperedens* archaea. Mini-Borg sequences fall with archaeal B6. Magro are viruses of marine Euryarchaeota. Borg sequences do not appear to share a recent common ancestor with *Nucleocytoviricota* but rather place firmly with sequences from Archaea. The tree is provided in **Supplementary data 20**.

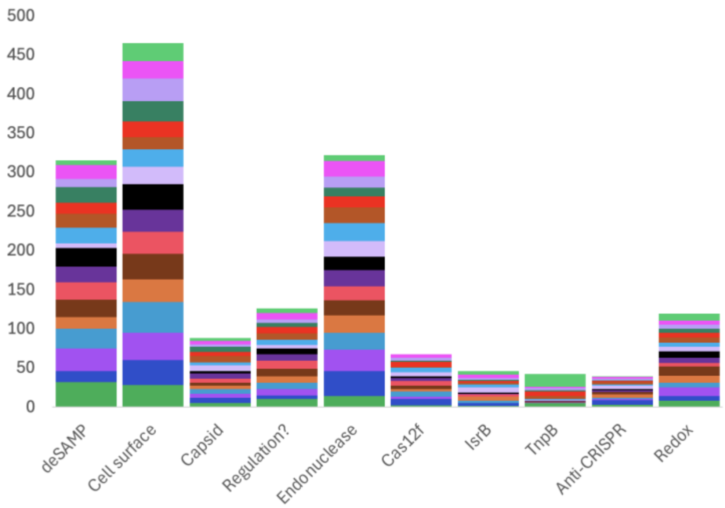

Figure S11: Overview of the more prevalent Borg multicopy proteins (**Supplementary Data 9**), in some cases groups of subfamilies, into categories based on related functions.

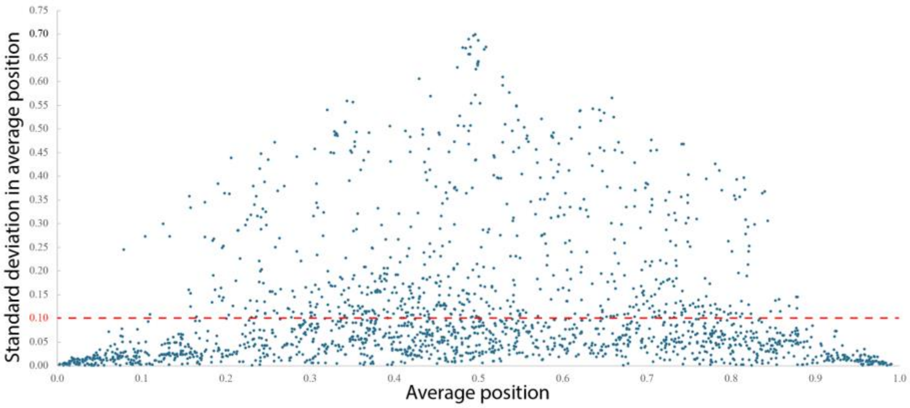

Supplementary Figure 12: A plot of the average gene position (x-axis) for each subfamily that is not in multicopy in any genome (1931 of 2609 subfamilies) vs. the standard deviation in this position (y-axis). 1243 (64%) of proteins have standard deviation values are ≤ ± 0.1 (dashed red line). 24% of cases with standard deviation values of > ± 0.1 are subfamilies with only two members.

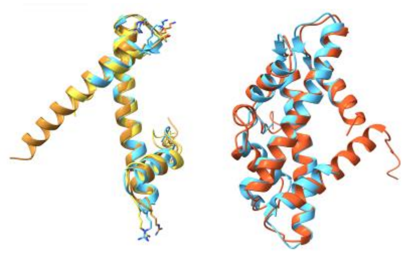

Supplementary Figure 13: **Borg Histones. A.** Consecutive histone-like proteins from Amethyst Borg are structurally similar (811 and 812, gold and orange), with best match to the experimentally characterized structure of a histone from *Pyrococcus horikoshii* (PDB 1KU5, blue). **B.** Amethyst 811 and 812 form a heterodimer (ipTM = 0.88, pTM = 0.86, red) that aligns well with that of 1KU5 (blue). Related sequences occur in *Methanoperedens* and phylogenetically intermix with Borg sequences.

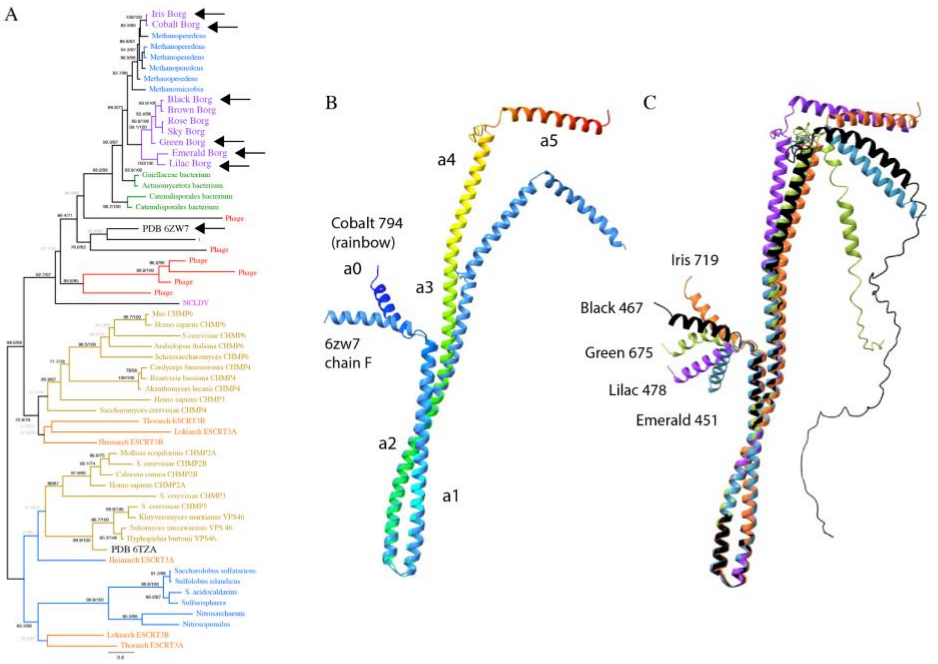

Supplementary Figure 14: Borg genomes encode membrane remodeling machinery. Among various proteins predicted to have functions analogous to those of the eukaryotic ESCRT system are a subset with structures analogous to that of bacterial membrane remodeling proteins exemplified by PDB 6zw7. These proteins share a common ancestor with the ESCRT III system of Eukaryotes (Spang et al. 2015). **A.** Phylogenetic tree (MAFFT alignment, trimmed using trimal gt 01) including 6zw7, Borg sequences and related sequences from bacteria (green font), archaea other than Asgard (blue font), Asgard archaea (orange font) and Eukaryotes (gold font). Arrows indicate the sequences used in structure predictions in B. and C. Interestingly, the Borg proteins are placed with those from *Methanoperedens* and related archaea, bacteria, and bacteriophages, but not with eukaryotic sequences. **B.** Alignment of the predicted structure of a protein from Cobalt Borg and a subunit of the 6zw7 homo-multimer. The Borg protein is rainbow colored from start (blue) to end (red) and labeled with the region designations analogous to those of ESCRT-III proteins. **C.** A subset of other Borg proteins grouped (based on sequences) into the same subfamily and predicted to have structures analogous to those of 6zw7 and other ESCRT - III proteins.

Supplementary References

1. Hug, L. A. *et al.* Community genomic analyses constrain the distribution of metabolic traits across the Chloroflexi phylum and indicate roles in sediment carbon cycling. *Microbiome* **1**, 22 (2013).
2. Gandini, R., Reichenbach, T., Tan, T.-C. & Divne, C. Structural basis for dolichylphosphate mannose biosynthesis. *Nat. Commun.* **8**, 120 (2017).
3. Taylor, Z. W., Brown, H. A., Holden, H. M. & Raushel, F. M. Biosynthesis of nucleoside diphosphoramidates in Campylobacter jejuni. *Biochemistry* **56**, 6079–6082 (2017).
4. Lindås, A.-C., Karlsson, E. A., Lindgren, M. T., Ettema, T. J. G. & Bernander, R. A unique cell division machinery in the Archaea. *Proc. Natl. Acad. Sci. U. S. A.* **105**, 18942–18946 (2008).
5. Samson, R. Y., Obita, T., Freund, S. M., Williams, R. L. & Bell, S. D. A role for the ESCRT system in cell division in archaea. *Science* **322**, 1710–1713 (2008).
6. Ellen, A. F. *et al.* Proteomic analysis of secreted membrane vesicles of archaeal Sulfolobus species reveals the presence of endosome sorting complex components. *Extremophiles* **13**, 67–79 (2009).
7. Tarrason Risa, G. *et al.* The proteasome controls ESCRT-III-mediated cell division in an archaeon. *Science* **369**, eaaz2532 (2020).
8. Spang, A. *et al.* Complex archaea that bridge the gap between prokaryotes and eukaryotes. *Nature* **521**, 173–179 (2015).
