# Supplementary material for: Convergent evolution of viral-like Borg archaeal extrachromosomal elements and giant eukaryotic viruses
Source: Nat Commun. 2025 Nov 27;16:10641. doi: 10.1038/s41467-025-65646-7 (PMC12660748; doi:10.1038/s41467-025-65646-7)
Supplement: Supplementary file 2 — Description of Additional Supplementary Files [file 41467_2025_65646_MOESM2_ESM.pdf]

Description of Additional Supplementary Files for:

File Name: Supplementary Data 1.
Type/format: ZIP of structures
Location: Zenodo: https://zenodo.org/records/15795806
Description: **A.** Highest confidence predicted structures of all folded proteins for seven Borgs in .pdb format. **B.** Full AlphaFold2 output for all of the 8847 Borg protein structures: https://zenodo.org/records/15795806 **C.** Predicted structures for featured proteins from other Borgs, e.g., putative deSAMPs from all Borgs, Sky 263, Apricot packaging ATPase, Purple 326 and Olive Borg, *Methanoperedens, Methanoregula* DPMS proteins with extra domains. **D.** Confidence plots (amino acid specific plDDT scores) for all folded proteins in **A.**

File Name: Supplementary Data 2.
Type/format: Excel (.xlsx)
Location: Provided with the article
Description: Overview of statistics for protein structure prediction confidence and bitscores at thresholds, with protein by protein listing of details including bitscore for each PDB match and PDB target model, PDB model header and mean, median pLDDT scores.

File Name: Supplementary Data 3 - 8.
Type/format: Excel (.xlsx)
Location: Provided with the article
Description: Listings of the identified the best matches for each protein with a structure predicted using AlphaFold 2 and in the PDB, along with bitscores and e-values, for seven Borgs.

File Name: Supplementary Data 9.
Type/format: Excel (.xlsx)
Location: Provided with the article
Description: Subfamily counts across the 17 Borg genomes, in some cases grouped by similar functions (blue highlights).

File Name: Supplementary Data 10.
Type/format: Tree file and iQTREE outputs
Location: Zenodo: https://zenodo.org/records/15795806
Description: Phylogenetic tree of putative deSAMPylases showing intermixing proteins from different subfamilies (iqTree, best-fit model: JTTDCMut+F+G4). Nodes with >0.75 bootstrap support are labeled.

File Name: Supplementary Data 11.
Type/format: MSA files
Location: Zenodo: https://zenodo.org/records/15795806
Description: **A.** Protein multisequence alignment for Borg, Methanoperedens, and other archaeal putative deSAMPylases. **B.** Protein multisequence alignment for Borg, Methanoperedens, and other archaeal putative SAMPs.

File Name: Supplementary Data 12.
Type/format: ZIP of structures
Location: Zenodo: https://zenodo.org/records/15795806
Description: All AlphaFold3 data for multimers, including five model structures (ranks 0- 4), PAE results and confidence metrics.

File Name: Supplementary Data 13.
Type/format: Excel (.xlsx)
Location: Provided with the article
Description: The ipTM values for all five models of example multimers involving the *Methanoperedens* SAMP and putative deSAMPylase enzymes, with the average value and standard deviation per multimer.

File Name: Supplementary Data 14.
Type/format: Tree file and iQTREE outputs
Location: Zenodo: https://zenodo.org/records/15795806
Description: Phylogenetic tree of putative DPMS (iqTree, best-fit model: JTTDCMut+F+G4). Nodes with >0.75 bootstrap support are labeled.

File Name: Supplementary Data 15.
Type/format: Excel (.xlsx)
Location: Provided with the article
Description: Comparison of single jelly roll (SJR) fold proteins with cellular and viral proteins From Kuprovic and Koonin (Figure 2: *https://www.pnas.org/doi/full/10.1073/pnas.1621061114*), reporting sequence alignment scores, RMSD over the number of pruned atom pairs and reference data information.

File Name: Supplementary Data 16.
Type/format: ZIP of structures
Location: Zenodo: https://zenodo.org/records/15795806
Description: Predictions of multimers of capsid-like proteins. For multimers with a range of composition, ipTM for the five models for each protein and pTM values, as well as structure configurations are provided. For the best model, the RMSD over pruned atom pairs and all pairs are provided, as well as the buried area between multimer subunits (as a measure of confidence of interaction).

File Name: Supplementary Data 17.
Type/format: Excel (.xlsx)
Location: Provided with the article
Description: The 17 Borg genomes have one to three genomic regions that encode at least one structural protein (listed by gene number) and occur in a reasonably well defined region of the genomes. The first region is highly expressed, see **Figure 5A**. The proteins in the second region have distant but detectable similarity to bacterial capsid-like / tail-like proteins. The third region features capsid-like proteins featuring a jelly roll fold, encoded consecutively or near-consecutively. These occur in a fairly confined and consistent relative position in each genome. For example, in Green Borg, which has 1517 protein-encoding genes, the three regions occur between gene 1003 and 1101. Genes that were assigned to subfamilies, as noted in different color text: brown = subfam1122, orange = subfam1116, aqua = subfam1011, green = subfam0857, rose pink = subfam2017, blue = subfam1406, purple = subfam1657, red = subfam0634, lime subfam1836.

File Name: Supplementary Data 18.
Type/format: MSA files
Location: Zenodo: https://zenodo.org/records/15795806
Description: Protein multisequence alignment for putative tail knob proteins.

File Name: Supplementary Data 19.
Type/format: Excel (.xlsx)
Location: Provided with the article
Description: Detailed version of the **Table 1** overview of the large inventory of genes that are normally only found in organisms and sometimes in giant eukaryotic viruses.

File Name: Supplementary Data 20.
Type/format: Tree file and iQTREE outputs
Location: Zenodo: https://zenodo.org/records/15795806
Description: Phylogenetic tree for DNA polymerase B (iqTree, best-fit model: JTTDCMut+F+G4). Nodes with >0.75 bootstrap support are labeled.

File Name: Supplementary Data 21.
Type/format: Excel (.xlsx)
Location: Provided with the article
Description: Listing of tandem repeat details for a set of publicly available *Nucleocytoviricota* genomes. Included are columns reporting sequence ID, Order, Genome name, Genome length, GC content, and statistics describing the instances of tandem repeat regions in these genomes. Results are provided for 65 genomes > 500 kbp and for diverse genomes > 10 kbp in length (including fragments). Taxonomic breakdown excluding consideration of fragments is provided in the main text. Extracted data (averages, standard deviations) are listed in **Table 2**.

File Name: Supplementary Data 22.
Type/format: Excel (.xlsx)
Location: Provided with the article
Description: Listing, per *Nucleocytoviricota* genome, of the repeat details statistics including the location of the repeat loci, the lengths of the unit repeats, number of repeat units per locus and the repeat sequence. Extracted data (averages, standard deviations) are listed in **Table 2**.

File Name: Supplementary Data 23.
Type/format: Excel (.xlsx)
Location: Provided with the article
Description: Listing of tandem repeat details for 17 Borg genomes, including genome length, GC content, and statistics describing the instances of tandem repeat regions in these genomes. Extracted data (averages, standard deviations) are listed in **Table 2**.

File Name: Supplementary Data 24.
Type/format: Excel (.xlsx)
Location: Provided with the article
Description: Listing, per Borg genome, of the repeat details statistics including the location of the repeat loci, the lengths of the unit repeats, number of repeat units per locus and the repeat sequence. Extracted data (averages, standard deviations) are listed in **Table 2**.

File Name: Supplementary Data 25.
Type/format: Excel (.xlsx)
Location: Provided with the article
Description: Gene numbers for putative and possible deSAMPylases in 17 genomes, indicative of their genomic distribution, as well as subfamily assignments (if available). Median pLDDT scores for proteins from the 7 Borg genomes analyzed in detail are provided.

File Name: Supplementary Data 26.
Type/format: Excel (.xlsx)
Location: Provided with the article
Description: Incidence of sequential genes from the same subfamily in the 7 Borg genomes analyzed in detail. Listing of a subfamily indicates two sequential proteins. Also provided is a list of the subfamilies that often occur sequentially in four or more Borg genomes and, predicted functions (if available) based primarily on structural information.

File Name: Supplementary Data 27
Type/format: Tree file and iQTREE outputs
Location: Zenodo: https://zenodo.org/records/15795806
Description: Phylogenetic tree for ESCRT-like proteins.
