# Supplementary material for: Convergent evolution of viral-like Borg archaeal extrachromosomal elements and giant eukaryotic viruses
Source: Nat Commun. 2025 Nov 27;16:10641. doi: 10.1038/s41467-025-65646-7 (PMC12660748; doi:10.1038/s41467-025-65646-7)
Supplement: Supplementary file 4 — Reporting Summary [file 41467_2025_65646_MOESM4_ESM.pdf]

nature portfolio

Corresponding author(s): Banfield and Knott

Last updated by author(s): 20225/09/09

Reporting Summary

Nature Portfolio wishes to improve the reproducibility of the work that we publish. This form provides structure for consistency and transparency in reporting. For further information on Nature Portfolio policies, see our Editorial Policies and the Editorial Policy Checklist.

Statistics

For all statistical analyses, confirm that the following items are present in the figure legend, table legend, main text, or Methods section.

n/a	Confirmed	
☒	☐	The exact sample size (*n*) for each experimental group/condition, given as a discrete number and unit of measurement
☒	☐	A statement on whether measurements were taken from distinct samples or whether the same sample was measured repeatedly
☒	☐	The statistical test(s) used AND whether they are one- or two-sided<br>*Only common tests should be described solely by name; describe more complex techniques in the Methods section.*
☒	☐	A description of all covariates tested
☒	☐	A description of any assumptions or corrections, such as tests of normality and adjustment for multiple comparisons
☒	☐	A full description of the statistical parameters including central tendency (e.g. means) or other basic estimates (e.g. regression coefficient) AND variation (e.g. standard deviation) or associated estimates of uncertainty (e.g. confidence intervals)
☐	☒	For null hypothesis testing, the test statistic (e.g. *F*, *t*, *r*) with confidence intervals, effect sizes, degrees of freedom and *P* value noted<br>*Give P values as exact values whenever suitable.*
☐	☒	For Bayesian analysis, information on the choice of priors and Markov chain Monte Carlo settings
☒	☐	For hierarchical and complex designs, identification of the appropriate level for tests and full reporting of outcomes
☒	☐	Estimates of effect sizes (e.g. Cohen's *d*, Pearson's *r*), indicating how they were calculated

Our web collection on statistics for biologists contains articles on many of the points above.

Software and code

Policy information about availability of computer code

Data collection: *Provide a description of all commercial, open source and custom code used to collect the data in this study, specifying the version used OR state that no software was used.*

Data analysis	Geneious (v11.0.18+10) Licensed, paid version used in this study, free versions available)<br>ggKbase (https://ggkbase.berkeley.edu/)<br>Prodigal (v2.6.3)<br>InterProScan (5.50-84.0)<br>AlphaFold2 (ColabFold v1.5.5)<br>AlphaFold3 (AlphaFold v3.0.)<br>Dali<br>FoldSeek v9<br>Repeatfinder (2023)<br>Tandem Repeat Finder<br>Glimmer v3.02<br>IQ-TREE v2.3.6<br>MAFFT v7.526<br>TrimAL v1.5.0<br>Adobe Illustrator

For manuscripts utilizing custom algorithms or software that are central to the research but not yet described in published literature, software must be made available to editors and reviewers. We strongly encourage code deposition in a community repository (e.g. GitHub). See the Nature Portfolio guidelines for submitting code & software for further information.

Data

Policy information about availability of data

All manuscripts must include a data availability statement. This statement should provide the following information, where applicable:

- Accession codes, unique identifiers, or web links for publicly available datasets
- A description of any restrictions on data availability
- For clinical datasets or third party data, please ensure that the statement adheres to our policy

As noted in Schoelmerich et al. 2024, the 17 Borg and Methanoperedens genomes are available via: https://ggkbase.berkeley.edu/borgs_mp_nanopore/organisms and have been deposited in the NCBI database under accession code PRJNA1119519. Source Data provided, are comprised of 20 Supplementary Tables, all predicted structures for monomers and multimers, multiprotein sequence alignments and phylogenetic trees. Other data reported in this study can be accessed via https://figshare.com/projects/Convergent_evolution_of_viral-like_Borg_archaeal_extrachromosomal_elements_and_giant_eukaryotic_viruses/255005

Research involving human participants, their data, or biological material

Policy information about studies with human participants or human data. See also policy information about sex, gender (identity/presentation), and sexual orientation and race, ethnicity and racism.

Reporting on sex and gender	NA
Reporting on race, ethnicity, or other socially relevant groupings	NA
Population characteristics	NA
Recruitment	NA
Ethics oversight	NA

Note that full information on the approval of the study protocol must also be provided in the manuscript.

Field-specific reporting

Please select the one below that is the best fit for your research. If you are not sure, read the appropriate sections before making your selection.

☐ Life sciences ☐ Behavioural & social sciences ☒ Ecological, evolutionary & environmental sciences

For a reference copy of the document with all sections, see nature.com/documents/nr-reporting-summary-flat.pdf

Ecological, evolutionary & environmental sciences study design

All studies must disclose on these points even when the disclosure is negative.

Study description	Analysis of publicly available data, primarily using in silico structural biology methods.
Research sample	Metagenomically derived complete and near-complete genomes.
Sampling strategy	We identified proteins that occur most frequently in the genomes.

Data collection	NA
Timing and spatial scale	Samples from the original published study were collected from one site over time and a range of soil depths.
Data exclusions	Structural models that received extremely low confidence scores (median pLDDT < 27) were excluded from consideration.
Reproducibility	Some AF3 calculations were run multiple times and lack of reproducibility for high multimers is noted in the manuscript.
Randomization	NA
Blinding	NA

Did the study involve field work? ☐ Yes ☒ No

Reporting for specific materials, systems and methods

We require information from authors about some types of materials, experimental systems and methods used in many studies. Here, indicate whether each material, system or method listed is relevant to your study. If you are not sure if a list item applies to your research, read the appropriate section before selecting a response.

Materials & experimental systems

n/a	Involved in the study
☒	☐ Antibodies
☒	☐ Eukaryotic cell lines
☒	☐ Palaeontology and archaeology
☒	☐ Animals and other organisms
☒	☐ Clinical data
☒	☐ Dual use research of concern
☒	☐ Plants

Methods

n/a	Involved in the study
☒	☐ ChIP-seq
☒	☐ Flow cytometry
☒	☐ MRI-based neuroimaging

Plants

Seed stocks	NA
Novel plant genotypes	NA
Authentication	NA
